# The effects of local variation in light availability on pollinator visitation, pollen and resource limitation of female reproduction in *Hosta ventricosa*

**DOI:** 10.1186/s40529-017-0180-z

**Published:** 2017-06-02

**Authors:** Guo-Xing Cao, Bi-Xian Wu, Xu-Jian Xu, Xie Wang, Chun-Ping Yang

**Affiliations:** 1 Department of Forestry, Sichuan Agricultural University, Chengdu, 611130 China; 2 Xishuangbanna Tropical Botanical Garden, Chinese Academy of Sciences, Mengla, 666303 China; 3 Soil and Fertilizer Research Institute, Sichuan Academy of Agricultural Sciences, Chengdu, 610066 Sichuan China

**Keywords:** *Hosta ventricosa*, Local light environment, Pollen limitation, Pollinator visitation rate, Resource limitation

## Abstract

**Background:**

Light availability may have direct effects on reproduction through resource availability, and indirect effects on female reproduction by influencing plant-pollinator interactions. Floral display size, pollinator visitation per flower, resource and pollen limitation of fruit and seed production were quantified in a forested patch and an adjacent open patch of two populations of the perennial herb *Hosta ventricosa*.

**Results:**

Plants in the open patch produced significantly larger floral displays than those in the forested patch in both populations. Floral display size had a positive effect on pollinator visitation rate per flower in one population, but no effect in the other. Plants in the open patch received approximately 8–11 times more visitation rates and produced significantly more fruit and seeds per flower than those in the forested patch. However, supplemental pollination resulted in significantly more fruit and seed production per flower compared to natural pollination in the forested patch but not in the open patch in one population, and did not enhance fruit and seed production in either the forested or the open patch in the other. In both populations, supplementally pollinated plants in the open patch produced significantly more fruit and seeds per flower than supplementally pollinated plants in the forested patch.

**Conclusions:**

In *H. ventricosa*, local variation in light conditions could affect pollinator activity and influence female reproduction through resource availability; however differences in the degree of pollen limitation between local habitats were found to be population-specific.

## Background

It is commonly observed that not all flowers produce fruit and not ovules in a flower mature seeds in flowering plants (Stephenson 1981; Wiens 1984; Sutherland 1986; Lee 1988). Two main proximate limiting factors, pollen limitation and resource limitation, have been proposed to explain these common phenomena (Haig and Westoby 1988). Pollen limitation, because of insufficient pollinator service, is usually tested by the addition of supplemental pollen to stigmas experimentally; reproduction of plants is considered pollen-limited if fruit or seed set is elevated by hand pollinations relative to natural pollinations (Burd 1994; Larson and Barrett 2000; Ashman et al. 2004; Knight et al. 2005; Wesselingh 2007). Resource limitation can be tested by the comparison of fruit and seed production between plants of different size (Griffin and Barrett 2002) or plants grown at different levels of physical resources (Lee and Bazzaz 1982).

Light availability is potentially an important resource that may constrain reproduction, because light intensity can affect net photosynthetic rate (Kitajima 1994). Experimental studies have shown that overall plant size is higher for plants grown with sun exposure compared to those grown in shade (Cai 2011; Zhao et al. 2012). Light environment can also affect the pattern of resources allocated to reproductive components; plants in low light environments have been shown to divert resources away from reproductive components to parts which can increase light capture, such as stems and leaves (McConnaughay and Coleman 1999). As a result of reductions in plant size and/or relative allocation to reproduction, flower and fruit production and the number of seeds that are matured may decrease with decreasing light availability (Niesenbaum 1993; Cunningham 1997; Kato and Hiura 1999; Cao and Kudo 2008).

Light availability may affect the degree of pollen limitation by plant-pollinator interactions and thus female reproduction indirectly. Because insect thermoregulatory capacity is higher in open than shaded habitats, pollinator visitation rate to flowers may be greater in the former (Herrera 1997; Kilkenny and Galloway 2008). Light conditions can also induce changes in floral display size (number of flowers open at one time on a plant), and thus may indirectly affect visitation rate to flowers. Plants occur in better light environments may produce larger floral displays, and pollinator visitation rate per flower have been shown to increase with increasing floral display size in some studies (Klinkhamer et al. 1989; Grindeland et al. 2005; Kilkenny and Galloway 2008). These differences in visitation rate between habitats, if large enough, could translate into greater pollen limitation of fruit and seed production in shaded habitats unless female reproduction of plants in shaded habitats is resource limited.

Plants of the same species often occur in locally contrasting light environments. When variation in light conditions is sufficient, female reproductive success may be directly affected by light availability, as well as indirectly affected by plant-pollinator interactions. In the present study, variations in pollinator visitation rate, pollen and resource limitation of female reproduction were simultaneously evaluated in the perennial animal-pollinated herb *Hosta ventricosa*, which grows in adjacent forested patches and open patches. Such simultaneous investigation could partition direct effects of light availability on plant reproduction through reproductive resource from indirect effects through changes in pollinator visitation rates. Specifically, the following questions were addressed: (1) Is pollinator visitation rate lower in forested patches than in open patches? (2) And if so, is the degree of pollen limitation of fruit and seed production greater in forested patches than in open patches? (3) Is fruit and seed production lower in forested patches than in open patches in the absence of pollen limitation?

## Methods

### Study species and populations

*Hosta ventricosa* Stearn (Hostaceae), a natural tetraploid, can reproduce via seeds and also vegetatively via rhizomes. Seeds are more commonly produced by pseudogamous apomixis, and less frequently through sexual reproduction (Schmid 2010). The species is cultivated widely in China and western countries not only for its ornamental value, but also for its consideration as a medicinal herb and economic value as a food source (Schmid 2010). *H. ventricosa* is a perennial hermaphroditic plant that grows in a variety of habitats, including forest floors and edges, along the edges of roadways, and in open areas (Schmid 2010; Cao et al. 2011). Individual plants produce several ramets and form small clone patches. Each ramet produces a single stem that bears several leaves at the base. In the study area in southern China, leaves emerge in early April and generally mature in mid-May. Bolting of flowering stems begins in early June. Six to greater than 30 flowers may be produced within a single raceme inflorescence. Commonly, one to three flowers open on the same day within an inflorescence. Individual flowers produce, on average, more than 30,000 pollen grains and approximately 40 ovules (Cao et al. 2015). The longevity of individual flowers is 1 day. *H. ventricosa* is adichogamous and seed set of self- and cross-pollinated flowers does not differ (Cao et al. 2015). Therefore, the species is fully self-compatible, although pollinator visitation is necessary for pollination. Bumblebees are the major pollinators of the species, and fruits mature at the end of September.

The experiments in the present study were conducted in one population (30°04′10′′N; 102°59′23′′E; At 990 m) in the Bifengxia National Nature Reserve, Yaan, Sichuan Province (hereafter, BYS), and one population (29°06′29′′N; 107°19′20′′E; At 1320 m) at Shanwangping Forest Farm, Nanchuan, Chongqing Municipality (hereafter, SNC), in 2013. Population BYS consisted of two relatively discontinuous patches, one located in the understory of an evergreen broadleaved forest, and the other in the open field. The forested patch was approximately 80 m^2^ in area, comprised 107 individuals, and an individual plant produced an average of 2.9 ± 1.0 (SD) flowering racemes. The open patch was approximately 130 m^2^ in area, comprised 84 individuals, and an individual plant produced an average of 3.3 ± 1.1 flowering racemes. The two patches were approximately 20 m apart. Relative light intensity was 13.2% in the forest at the flowering season. Flowering commenced in mid-June, and lasted until mid-July. Population SNC was approximately 500 m^2^ in area, comprised more than five hundred individuals, and each individual produced an average of 2.5 ± 0.7 flowering racemes. In addition, approximately 60% of the individuals were located in the understory of an evergreen and deciduous broadleaved mixed forest, and the remaining plants were located in the open field. Relative light intensity was 10.8% in the forest at the flowering season. Flowering commenced in early July and lasted until late July. In all experiments conducted in population SNC, plants growing in an approximately 10-m border zone between the forest and open field were eliminated from the study.

### Pollinator visitation

Pollinator visitation was observed over four separate sunny days for each population. Observations were conducted in late June and mid-July, 2013, at the peak flowering of populations BYS and SNC, respectively. Each day before observation, 28–32 flowering racemes were randomly tagged at each of the forested and open patch, and the number of open flowers on each tagged flowering raceme was counted. Between 9:00 a.m. and 15:00 p.m. each day, eight 15-min observation periods were carried out at each of the two patches. During each 15-min observation period, three separate observers each observed approximately 10 tagged racemes. Each round of observation was alternated between the two patches within a single day. All pollinator visits to individual open flowers of each tagged raceme were recorded. Visitation rate was calculated as the number of visits per flower per hour.

### Pollen and resource limitation

To evaluate whether fruit and seed production were limited by pollen availability, as well as whether pollen limitation differed between the forested patch and the open patch, forty experimental plants were randomly selected at each of the two patches before flowering in each population. Two racemes were randomly tagged from each experimental plant, and one as an experimental raceme and was given supplemental pollen by hand pollination, while the other served as a control raceme and received only natural pollination. For each supplemental raceme on experimental plants, all of the flowers were hand-pollinated daily for each population, with the exception of 2–3 rainy days. Pollen was collected from plants 3 m away from the recipient plant. The hand-pollinated flowers were subsequently exposed to open pollination. In addition, in order to test whether experimental plants could reallocate resources away from control racemes to supplemental racemes, thirty-five control racemes from neighboring control (i.e., unmanipulated) plants at similar flowering stage and similar inflorescence size were randomly tagged at each of the two patches before flowering in each population. All of the infructescences were collected after the fruits had matured, the number of mature fruit or flower scars on each raceme was recorded, and the number of mature seeds in each fruit was assessed.

### Statistical analyses

All analyses were carried out using the open-source software R (version 3.32, R Development Core Team 2014). To test the effects of habitat on display size, the lmer function of the ‘lme4’ package and a Poisson error distribution were used. The model included habitat (forested and open), population (BYS and SNC), and their interaction as fixed factors, and day was included as a random factor. Significance of fixed effects was examined through *F*-tests using the procedure ‘anova’ in R.

To test for effects of habitat and display size on pollinator visitation rate to flowers, the lmer function of the ‘lme4’ package and a Poisson error distribution were also used. The model included habitat, display size, and their interaction as fixed factors, and day was included as a random factor. Initially, population was included as a fixed factor, however, because of significant interactions of main effects with population, the two populations were analyzed separately.

Evidence for pollen limitation was assessed by comparing fruit per flower and seeds per flower between the control raceme on experimental plants and the supplemental raceme on experimental plants. Effects of habitat, pollination treatment (natural and supplemental), and their interaction on the two reproductive variables were tested using the lmer function of the ‘lme4’ package and a Gaussian error distribution. The model included habitat, pollination, and their interaction as fixed factors, and plant was included as a random factor. Initially, population was included as a fixed factor, however, because of significant interactions of main effects with population, the two populations were analyzed separately.

Resource limitation was assessed by testing the effects of habitat on fruit and seed production using only supplemental racemes on experimental plants. The lm function of the ‘stats’ package and a Gaussian error distribution were used, with habitat as a fixed factor.

To test for the possibility of reallocation of resources to supplementally pollinated racemes within plants, fruit and seed production were compared between the control raceme on experimental plants and the control raceme on control plants using the lm function of the ‘stats’ package and a Gaussian error distribution. The model included treatment, habitat, population, and all two-way and three-way interactions between these parameters as fixed factors.

## Results

### Display size

Display size was 2.3 ± 0.6 (SD) and 2.6 ± 0.8 for the forested patch and the open patch, respectively, in population BYS, and was 1.9 ± 0.6 (SD) and 2.1 ± 0.6 for the forested patch and the open patch, respectively, in population SNC. The differences in display size observed between habitats were found to be statistically significant (Table 1).

**Table 1:** Results of the linear mixed model test of the effects of habitat (forested vs. open patch) and population on display size in *H*. *ventricosa*

Factor	*F*_1,476_	*P*
Habitat	27.886	<0.001
Population	52.219	<0.001
Population × habitat	0.000	0.998

### Pollinator visitation

**Fig. 1:**
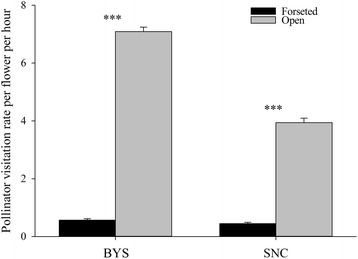
Total pollinator visitation rate per flower per hour (mean ± SE) in the forested and open patches in two populations of *H*. *ventricosa*. ***P < 0.001

During the 4-day observation in population BYS, the total number of approaches to tagged racemes was 184 for bumblebees, 39 for honeybees and two for butterflies in the forested patch, and 2370 for bumblebees, 516 for honeybees and six for butterflies in the open patch. The effect of habitat on total visitation rate per flower per hour was significant (Table 2); flowers in the open patch received 11 times more visitation rates than flowers in the forested patch (Fig. 1). Total visitation rate per flower were also found to vary with display size (Table 2); there was a positive correlation between visitation rate per flower (*Y*) and display size (*X*) in the forested patch (*Y* = 0.35*X* − 0.25, *R* = 0.46, *P* < 0.001) and in the open patch (*Y* = 0.42*X* − 5.98, *R* = 0.19, *P* < 0.01).

**Table 2:** Results of the linear mixed model test of the effects of habitat (forested vs. open patch) and display size on pollinator visitation rate per flower per hour in the two populations of *H*. *ventricosa*

Factor	BYS		SNC	
	*F*_1,236_	*P*	*F*_1,236_	*P*
Habitat	114.000	<0.001	36.089	<0.001
Display size	11.578	<0.001	0.037	0.848
Display size × habitat	0.083	0.773	0.001	0.981

During the 4-day observation in population SNC, the total number of approaches to tagged racemes was 162 for bumblebees and 22 for honeybees in the forested patch, and 1423 for bumblebees and 176 for honeybees in the open patch. Visitation rate per flower differed significantly between habitat, but did not vary with display size (Table 2). Flowers in the open patch received approximately eight times more visitation rates than flowers in the forested patch (Fig. 1).

### Pollen and resource limitation of female reproduction

**Fig. 2:**
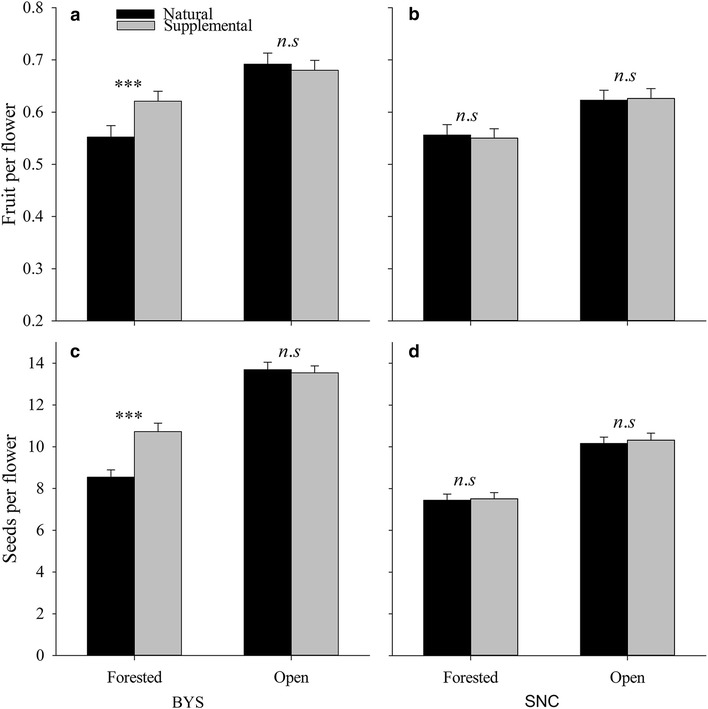
Fruit (**a**,** b**) and seeds (**c**,** d**) per flower (mean ± SE) for natural and supplemental pollination in the forested and open patches in two populations of *H*. *ventricosa*. *n*.*s* = not significant, ***P < 0.001

In population BYS, pollination treatment had significant effects on fruit and seed production, although a significant interaction term between pollination treatment and habitat was detected (Table 3), indicating that the effect of pollination treatment on fruit and seed production was dependent on habitat. In the open patch, pollination treatment did not affect either fruit per flower (*F*_1,39_ = 0.578, *P* = 0.452; Fig. 2a) or seeds per flower (*F*_1,39_ = 0.659, *P* = 0.385; Fig. 2c), while in the forested patch, pollination treatment significantly affected both fruit per flower (*F*_1,39_ = 50.167, *P* < 0.001) and seeds per flower (*F*_1,39_ = 111, *P* < 0.001); supplemental pollination increased fruit and seed production over natural pollination (Fig. 2a, c). Habitat also had a significant effect on female reproduction (Table 3). Supplementally pollinated flowers in the open patch produced more fruit per flower (*F*_1,78_ = 5.994, *P* = 0.017; Fig. 2a) and seeds per flower (*F*_1,78_ = 28.869, *P* < 0.001; Fig. 2c) than supplementally pollinated flowers in the forested patch.

**Table 3:** Results of the linear mixed model test of the effects of habitat (forested vs. open patch) and pollination treatment (natural vs. supplemental) on fruit and seed production in the two populations of *H*. *ventricosa*

Factor	BYS		SNC	
	*F*_1,78_	*P*	*F*_1,78_	*P*
Fruit per flower				
Habitat	21.207	<0.001	23.471	<0.001
Pollination	19.565	<0.001	0.114	0.737
Pollination × habitat	30.335	<0.001	0.001	0.983
Seeds per flower				
Habitat	80.301	<0.001	71.044	<0.001
Pollination	74.637	<0.001	0.640	0.426
Pollination × habitat	108.341	<0.001	0.100	0.753

In population SNC, neither the main effect of pollination treatment nor the interaction term between pollination treatment and habitat was significant (Table 3), and fruit per flower and seeds per flower were both similar for natural pollination and supplemental pollination in both the forested and open patches (Fig. 2b, d). Moreover, a significant effect of habitat was detected (Table 3). Supplementally pollinated flowers in the open patch produced more fruit per flower (*F*_1,78_ = 18.324, *P* < 0.001; Fig. 2b) and seeds per flower (*F*_1,78_ = 60.930, *P* < 0.001; Fig. 2d) than supplementally pollinated flowers in the forested patch.

### Resource reallocation

Control racemes on control plants in the forested and open patches in population BYS produced 0.56 ± 0.01 (SE) and 0.68 ± 0.01 fruit per flower, and 8.61 ± 0.27 and 13.41 ± 0.35 seeds per flower, respectively. Control racemes on control plants in the forested and open patches in population SNC produced 0.55 ± 0.01 and 0.62 ± 0.01 fruit per flower, and 7.38 ± 0.29 and 10.09 ± 0.24 seeds per flower, respectively. There was no difference observed in fruit production between control racemes on experimental plants and control racemes on control plants, as indicated by the lack of treatment effect (*F*_1,292_ = 0.929, *P* = 0.336), the interactive effects between treatment and habitat (*F*_1,292_ = 0.002, *P* = 0.964) and between treatment and population (*F*_1,292_ = 0.118, *P* = 0.732), and the three-way interactive effects between them (*F*_1,292_ = 0.067, *P* = 0.795). In addition, there was no difference in seed production between control racemes on experimental plants and control racemes on control plants, as indicated by the lack of treatment effect (*F*_1,292_ = 0.840, *P* = 0.360), the interactive effects between treatment and habitat (*F*_1,292_ = 0.033, *P* = 0.856) and between treatment and population (*F*_1,292_ = 0.190, *P* = 0.663), and the three-way interactive effects between them (*F*_1,292_ = 0.024, *P* = 0.877). These results indicated that there was no evidence of reallocation of resources from control racemes to experimental racemes within experimental plants.

## Discussion

### Effects of local habitat on pollinator visitation

*Hosta ventricosa* racemes in the open patch produced larger floral displays than racemes in the forested patch, a common result reflecting differences in overall plant resource status between patches (Kato and Hiura 1999; Meagher and Delph 2001). In population BYS, both light availability and floral display had positive effects on pollinator visitation rate per flower; in population SNC, however, only light availability had positive effects on pollinator visitation rate per flower. Therefore, indirect effects on visitation rate through display size in population BYS must be interpreted with some caution. For example, Kilkenny and Galloway (2008) reported that both light environment and floral display had positive effects on pollinator visitation rate per flower in *Campanulastrum americanum* plants growing in sunny and shady areas of a natural population, but visitation rate per flower responded positively to light levels and did not respond to display size in experimental arrays. Similar experimental arrays with different display sizes and light levels may verify whether floral display had an effect on visitation rate per flower in *H*. *ventricosa*.

Our results are consistent with those of Herrera (1997), who found that Hymenopterans preferred to forage in areas with high irradiance. Low light may be associated with lower visitation rate, if the flower color of *H*. *ventricosa* is less vibrant and therefore less visible in forested patches (Chittka and Raine 2006). Light availability might also correlate with visitation rate because of temperature (Corbet et al. 1993; Totland 2001). Since light availability has a positive effect on temperature (Herrera 1995; Kilkenny and Galloway 2008), it is likely that differences in visitation rate between forested patches and open patches could be due to both variations in light level and temperature.

### Variations in pollen and resource limitation between local habitat

In population SNC, pollen supplementation did not enhance fruit and seed production at the whole population level, indicating a lack of pollen limitation. These results are in accordance with those of Niesenbaum (1993) and Hasegawa and Kudo (2005) in other systems, who found that female reproduction in both sunny and shady areas was not limited by pollen. In population BYS, pollen supplementation increased fruit and seed production in the forested patch but not in the open patch, and there was no evidence of reallocation of resources to supplementally pollinated racemes, indicating that pollen limitation occurred in the forested patch but not the open patch, therefore differences in visitation rate between habitat influenced female reproduction. These results are consistent with those of Kilkenny and Galloway (2008) and Kudo et al. (2008) in other systems, who reported that a decline in light availability was a mechanism causing pollen limitation. Our study suggested that whether female reproduction in shady areas was limited by pollen availability is population-specific.

Light availability appears to limit reproduction in *H*. *ventricosa*. Fruit and seed production per flower for supplementally pollinated racemes were higher in the open patch than in the forested patch in both populations, results consistent with the findings of many studies (Niesenbaum 1993; Kato and Hiura 1999; Setsuko and Tomaru 2011), supporting the hypothesis that light availability could affect female reproduction through resource availability.

## Conclusion

The present study evaluated the effects of the local light environment on pollinator visitation, pollen and resource limitation of female reproduction in two populations of *H*. *ventricosa*. The results showed that light environment could directly influence pollinator behavior, although the relative importance of resource-mediated effect and pollinator-mediated effect on female reproduction may vary among populations.
